# Cochlear Implantation in Infants: Why and How

**DOI:** 10.1177/23312165211031751

**Published:** 2021-07-19

**Authors:** Patricia L. Purcell, Nicholas L. Deep, Susan B. Waltzman, J. Thomas Roland, Sharon L. Cushing, Blake C. Papsin, Karen A. Gordon

**Affiliations:** 1 Department of Otolaryngology, Head & Neck Surgery, The 7979 Hospital for Sick Children, Toronto, Ontario, Canada; 2 Department of Otolaryngology, Head & Neck Surgery, New York University Grossman School of Medicine, New York, New York, United States

**Keywords:** cochlear implantation, congenital hearing loss, profound hearing loss, infants

## Abstract

In children with congenital deafness, cochlear implantation (CI) prior to 12 months of age offers the opportunity to foster more typical auditory development during late infancy and early childhood. Recent studies have found a positive association between early implantation and expressive and receptive language outcomes, with some children able to achieve normal language skills by the time of school entry. Universal newborn hearing screening improved early detection and diagnosis of congenital hearing loss, allowing for earlier intervention, including decision-making regarding cochlear implant (CI) candidacy. It can be more challenging to confirm CI candidacy in infants; therefore, a multidisciplinary approach, including objective audiometric testing, is recommended to not only confirm the diagnosis but also to counsel families regarding expectations and long-term management. Surgeons performing CI surgery in young children should consider both the anesthetic risks of surgery in infancy and the ways in which mastoid anatomy may differ between infants and older children or adults. Multiple studies have found CI surgery in infants can be performed safely and effectively. This article reviews current evidence regarding indications for implantation in children younger than 12 months of age and discusses perioperative considerations and surgical technique.

In caring for children with congenital deafness, pediatric cochlear implant (CI) teams have witnessed the benchmark for age at implantation shift earlier and earlier, as providers have attempted to balance the need for surgical safety with the goal of providing early access to sound to the developing brain (Russell et al., 2013; Teagle et al., 2019; Yoon, 2011). In 2000, Food and Drug Administration (FDA) approval was provided for children as young as 12 months of age to receive CIs. Over the past 20 years, there has been increasing interest in the role of implantation in children younger than 12 months of age, with the goal of further reducing the gap in auditory development between children with congenital deafness and children with typical hearing (Luxford et al., 2004; Roland & Waltzman, 2015). In March 2020, the FDA provided device-specific approval for CI in infants with bilateral profound sensorineural hearing loss down to 9 months of age (U.S. Food and Drug Administration, 2020). Early implantation has been shown to improve auditory development, expressive and receptive language outcomes, and quality of life (Dettman et al., 2016b; Karltorp et al., 2020; Papsin & Gordon, 2007). This article reviews the current evidence regarding indications for implantation in children younger than 12 months of age and focuses on perioperative considerations and surgical technique.

## Auditory Development

In children with congenital deafness, implantation prior to 12 months of age offers the opportunity to foster auditory development during infancy and early childhood. The central auditory system develops through experience with sound, a process which is particularly active during time-sensitive developmental periods (Sanes & Bao, 2009; Werker & Tees, 2005). Sounds help to *map* the auditory cortex so that it reflects the organization of the frequency filters of the cochlea (Langers & van Dijk, 2012). The development of neural connections, a process known as functional synaptogenesis, peaks within the auditory cortex at around 2 to 4 years of age (Huttenlocher & Dabholkar, 1997). Synaptogenesis then slows over time, a phenomenon that supports the idea of a critical period for cochleotopic organization of the developing auditory cortex. The immature brain, with its increased plasticity, is especially suited to benefit from early cochlear implantation (O’Neil et al., 2011). In this way, access to sound and appropriate therapy during early childhood offers lifelong benefit to children with hearing loss (Kennedy et al., 2006). Children who receive a CI in infancy will benefit from auditory stimulation and demonstrate development in the brainstem and the thalamocortex (Gordon et al.,2003, 2005, 2006, 2008). However, if it is deprived of access to sound, the auditory cortex will experience arrest of auditory development and demonstrate cortical reorganization over time (Sharma et al., 2005). Importantly, children with bilateral deafness who receive only a unilateral implant will also demonstrate reorganization in the form of aural preference toward the implanted ear; there is evidence to support increasing asymmetry in access to sound the longer the delay between sequential implant placement (Gordon et al., 2011; Illg et al., 2019; Polonenko et al., 2018).

While studies of central auditory development reflect the importance of a sensitive period for cortical plasticity that peaks around 2 to 4 years of age, the functional importance of CI in children younger than the age of 12 months is probably best highlighted by studies specifically investigating expressive and receptive language outcomes. Comprehensive literature reviews of language development in children with congenital deafness suggest that early implantation may prevent long-term spoken language deficits in children implanted prior to 12 months of age (Ruben, 2018; Sharma et al., 2020). In one of the earliest comparative cohort studies examining this question, Colletti et al. (2011) studied a cohort of 19 children who had been implanted prior to 12 months of age, comparing their speech and language development over 10 years of follow-up to children implanted after 12 months of age. The authors found that, after 5 years of CI usage, all 19 children implanted prior to 12 months of age had achieved speech that was intelligible to the average listener, compared to only 67% of children implanted between 12 and 23 months of age. In addition, after 10 years of CI usage, all of the children implanted prior to 12 months of age received a grammar development score above the 75th percentile on the Test of Reception of Grammar, compared to only 38% of children implanted between 12 and 23 months of age. Dettman et al. (2016b) examined a cohort of more than 400 children with CI, finding that children implanted before 12 months of age demonstrated significantly better speech perception, language acquisition, and speech production accuracy; the children implanted prior to 12 months of age were also more likely to score within the normative range on language performance by the time of school entry. Bruijnzeel et al. (2016) performed a systematic review of studies investigating potential speech and language benefit for children who received CI surgery prior to 12 months of age. The authors identified 14 studies and found that children who were implanted before 12 months of age performed better on speech production, auditory performance, and some receptive language scores. Similarly, Ching et al. (2017) published the results of a prospective study of 350 children with hearing loss, finding a strong positive benefit to early intervention; for example, children implanted at 6 months had significantly higher global language scores (a summary score of 20 language measures) at 5 years of age than children implanted by 24 months. More recently, Mitchell et al.(2020) compared 29 children with congenital deafness who were implanted under 12 months with 82 children who had implant surgery between 12 and 24 months; they found that cochlear implantation under the age of 12 months was significantly associated with better performance on Preschool Language Scale – Auditory Comprehension assessment. Karltorp et al. (2020) found that children who were implanted during infancy experienced spoken language development that was more typical than children who were implanted after 12 months of age. The authors found that children who received their first CI younger than 12 months of age achieved age equivalency in language milestones and demonstrated better speech intelligibility, but did not have significantly better open-set word recognition, than children implanted after 12 months of age.

## The Role of Universal Newborn Hearing Screening

Early implantation has been associated with the implementation of universal newborn hearing screening (UNHS; Nelson et al., 2008). Children with congenital hearing loss often demonstrate typical motor and social development during infancy, thus masking any underlying hearing concern. In the absence of universal screening, diagnosis of hearing loss can be delayed until parents become aware that auditory development and spoken milestones are not being met (Thompson et al., 2001). With UNHS, approximately 1 in 1,000 newborns will receive referral for permanent congenital hearing loss (Butcher et al., 2019). UNHS lowered the average age at diagnosis of hearing loss, allowing for earlier intervention, including decision-making regarding CI candidacy, and ultimately improvement in expressive and receptive language outcomes (Dettman et al., 2016a; Yoshinaga-Itano et al., 2000).

The U.S. National Institutes of Health Consensus Development Conference (1993) released a consensus statement recommending implementation of UNHS for all children by 3 months of age.The availability of UNHS allowed for the Joint Committee on Infant Hearing of the American Academy of Pediatrics (2007) to establish the “1-3-6” guideline: that children undergo hearing screening by 1 month of age, complete diagnostic assessment by 3 months of age if needed, and receive early intervention by 6 months of age if they have hearing loss (Meinzen-Derr et al.,2020). For a child with congenital deafness, hearing amplification will not provide benefit; therefore, intervention requires adoption of sign language, cochlear implantation, or often a combination of both. In a recent executive summary update, Joint Committee on Infant Hearing (2019) encouraged programs that were meeting the 1-3-6 benchmark to now target a 1-2-3 month timeline for screening, diagnosis, and intervention, respectively; in addition, the summary emphasized the benefit of expanding the FDA-approved age for CI placement to infants younger than the age of 12 months.

Despite increasing awareness of the benefits of early access to sound, barriers remain to ensuring children with hearing loss are implanted in a timely fashion. In a 2015 population-based study of 187 children over 12 years, Fitzpatrick et al. (2015) found the most common reasons for delay in implantation to be the presence of progressive hearing loss (53%), complex medical conditions (17%), family indecision (9%), or geographic location (6%). An institutional study of 90 patients at Johns Hopkins University found that insurance status, race/ethnicity, and primary language other than English were associated with delays in diagnosis and intervention among children with hearing loss (Zhang et al., 2019); the authors hypothesize that these variables are likely proxies for lower socioeconomic status (SES). Lower SES, along with rural residency, has been associated with delays to cochlear implantation and worse outcomes following implantation in the literature (Noblitt et al., 2018). Additional research is needed to reduce loss to follow-up after newborn hearing screening (Razak et al., 2020) and improve identification of children who have risk factors for hearing loss (Beswick et al., 2013; Dumanch et al, 2017); such research may help to improve access to early intervention and reduce inequities in outcomes among children with hearing loss.

Factors such as familial environment and access to care that facilitate early diagnosis and intervention may also be associated with positive outcomes for language development (Duchesne & Marschark, 2019). Holzinger et al. (2020) found that parental linguistic input early in childhood was an important predictor of language development, accounting for more than 30% of variance in language outcomes. In the Carolina Sibling Study, Selleck et al. (2019) attempted to control for familial/environmental factors by looking for effect of age at implantation between sibling pairs implanted at different ages. With this analysis, they did not find an association between age at implantation and speech perception scores; however, the authors admit that the study was limited by small sample size. Tajudeen et al (2010) compared speech perception of children who were implanted in the first, second, or third years of life, identifying a benefit to implantation under 12 months due to attainment of high speech perception scores earlier in life; however, they did find all groups performed well over time, which can help to reassure families of children identified as CI candidates after 12 months of age. It would be beneficial for additional studies to investigate how parental education, SES, and home environment influence the effects of age at implantation on outcomes in infants who undergo CI surgery under 12 months of age. These factors should also be considered for additional outcome measures including cognitive, academic, and language assessments.

## Preoperative Assessment

**Figure 1.**
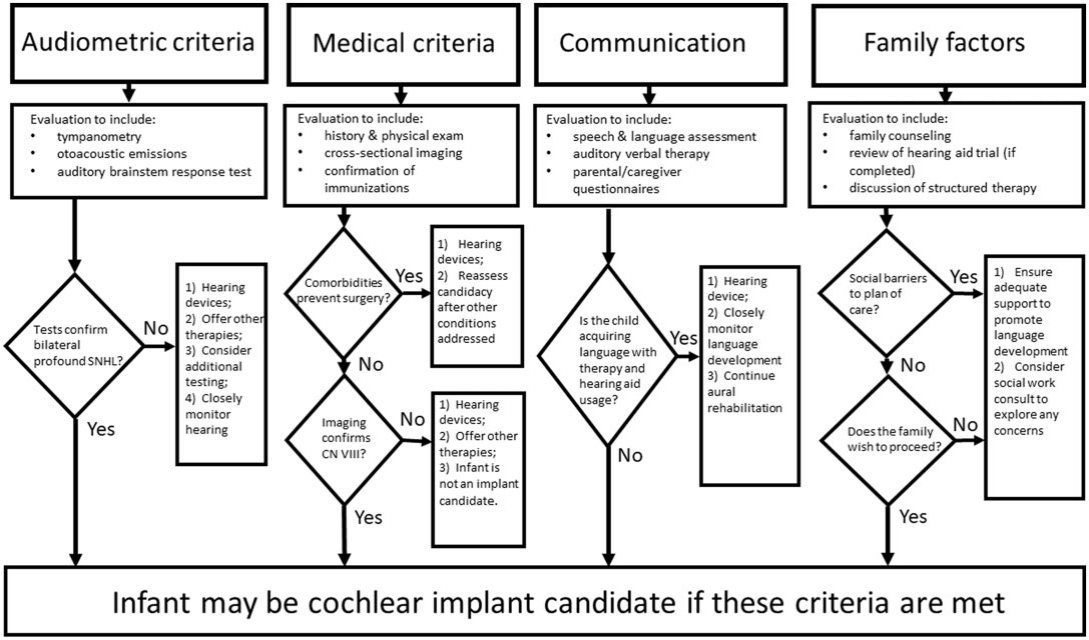
Criteria for Multidisciplinary Cochlear Implant Candidacy Assessment in Very Young Children. CN VIII = cranial nerve eight.

CI candidacy assessment is multidisciplinary and includes audiometric, medical, communication, and family/environmental components (Figure 1). The child should be confirmed to have bilateral profound hearing loss in which amplification does not provide adequate access to sound. Objective testing should include tympanometry, diagnostic auditory brainstem testing, and otoacoustic emission assessment (Cosetti & Roland, 2010a). In an infant, all tests should support a diagnosis of bilateral profound sensorineural hearing loss. If there are inconsistencies in the testing, then these should be addressed prior to any recommendation. Evidenced-based guidelines suggest that infants with a pure tone average better than 65 dB should continue with hearing aids, while infants with pure tone average poorer than 80 dB should proceed to CI. Multidisciplinary CI teams often must closely monitor infants whose hearing thresholds fall in between 65 and 80 dB HL (Leigh et al., 2016). The multidisciplinary CI team should include speech language pathologists and auditory verbal therapists, who are speech language pathologists, teachers, or audiologists who have received additional auditory verbal therapist training (Binos et al., 2021). They provide input on the child’s speech and language development based on evaluations suitable for assessment of infant patients (Percy-Smith et al., 2018; Thomas & Zwolan, 2019).Protocols for hearing aid fitting in infants with permanent hearing loss include monitoring of auditory and language development with parental report/questionnaires, along with therapy to promote language (Ganek et al.,2020). Teams should be cautious about proceeding to CI when infants are making progress in language development with consistent hearing aid use. Using a family-centered approach, a multidisciplinary CI team can help the child’s caregivers decide upon the best approach for language development, considering each family’s unique characteristics. While guidelines for etiologic workup of hearing loss are outside the scope of this review, identification of an etiology of deafness, such as congenital cytomegalovirus infection or genetic mutation, can help to inform decision-making and bolster confidence in the results of both objective and behavioral measures of hearing in infants (Lieu et al.,2020).

Preoperatively, the anatomy of the cochleovestibular structures and integrity of the cochlear nerve should be confirmed with imaging (Young et al., 2014). The details of preoperative imaging protocol are typically surgeon- and institution-dependent. One approach suggested in the literature is to perform magnetic resonance imaging (MRI) for all children undergoing assessment for cochlear implant candidacy and add computed tomography of temporal bone for patients considered at risk of having more surgically challenging anatomy, such as children with craniofacial anomalies (Chin et al.,2020; Siu et al., 2019; Trimble et al., 2007). However, some institutions prefer to recommend computed tomography for almost all children with congenital deafness who are potential CI candidates (Sepahdari et al., 2014; Yigit et al., 2019). The authors feel that MRI, in particular heavily weighted T2 sequences (CISS, SPACE, FIESTA), offer the most information about cochlear nerve size and integrity, unusual morphologic inner ear anatomy, and intracranial anomalies that might identify the etiology of deafness and possibly affect the cochlear implant process.

The child’s family should be counseled regarding the importance of structured therapy after surgery (Van Wieringen & Wouters, 2015). A hearing aid trial may be completed even if there is little expectation of auditory benefit (Houston & Bradham, 2021); the trial might provide an opportunity for the infant to adjust to wearing a device and for the family to become familiar with maintenance and care.Importantly, cochlear implantation should not be delayed to complete a hearing aid trial if a child has bilateral profound sensorineural hearing loss (American Academy of Otolaryngology–Head & Neck Surgery, 2020). The infant should also be up to date on immunizations, especially pneumococcal vaccine (Kahue et al., 2014). It can be difficult to counsel families regarding long-term expectations in children receiving implants under 12 months of age because developmental disorders, such as autism, may not be diagnosed until later in childhood (Hang et al., 2012). For a child with congenital profound deafness, hearing amplification may not provide access to the sounds of speech; thus, spoken language may be significantly delayed. Some, but not all, guidelines recommend early adoption of sign language, a decision to be made by parents after being fully informed (Caselli et al., 2021; Geers et al.,2017). Families should be informed of the potential for variability in speech and language outcomes despite early implantation. If families choose to proceed with early cochlear implantation in a child without comorbidities, the child will likely develop spoken language (Hoff et al., 2019) with long-term outcomes within the normative range for age (Miyamoto et al.,2017).

## Perioperative Considerations

Surgical considerations regarding the care of infants must take into account both anesthetic and anatomic concerns. First, CI surgery in infants should be performed in coordination with an experienced pediatric anesthesiology team. Anesthetic concerns specific to infancy include the fact that these patients are more prone to hypothermia (Lai et al., 2019) and have smaller blood volumes. An infant has an approximate blood volume of 75 ml/kg, so a 9-kg infant will have a total blood volume of only 675 ml (Howie, 2010). Therefore, 100 ml blood loss is equivalent to almost 15% of an infant’s total blood volume, which will be hemodynamically significant. Research is ongoing regarding the risk of anesthesia-induced neurotoxicity in infancy (Vutskits & Davidson, 2017). There is high-level evidence to suggest that anesthetic up to 1 hour in duration is not associated with long-term adverse effects on neurodevelopment (Grabowski et al., 2021; McCann et al., 2019); however, the effects of longer durations of general anesthesia are less clear. It is also unclear whether multiple exposures pose a greater risk than a single exposure (Davidson & Sun, 2018). Surgeons can attempt to minimize the duration of exposure to general anesthesia in the infant patient by avoiding sedated procedures when possible and by combining procedures such as MRI and auditory brainstem response under the same anesthetic if necessary.It has also been reported that simultaneous bilateral implantation is associated with a shorter total duration of general anesthesia when compared to sequential implantation (Uecker et al., 2019).

From an anatomical perspective, infants have a smaller mastoid bone with higher marrow content, and an underdeveloped mastoid tip places the facial nerve at increased risk of injury (James & Papsin, 2004a, 2004b). Despite these differences, several studies have found that infant implantation is safe and effective for the experienced surgeon (McKinney,2017; O’Connell et al., 2016; Roland et al., 2009; Valencia et al., 2008), a finding consistent with the multi-institutional retrospective study of safety outcomes in infant implantation that accompanies this review article (Deep et al., 2021).

While some variations in surgical technique have been reported in the literature regarding CI surgery in young children, the general approaches that have been described are similar. Facial nerve monitoring should be available and confirmed prior to the start of the case. A skin incision of approximately 3 cm, either postauricularly or more posteriorly placed along the hairline (Davids et al., 2009; James & Papsin, 2004a, 2004b; O’Donoghue & Nikolopoulos, 2002), allows sufficient access while also minimizing soft tissue disruption. In addition, care must be taken to avoid compromising the integrity of the musculoperiosteal Palva flap, typically a *U-shaped* anteriorly-based flap (Palva, 1963), which can be quite thin in infants. Because of the more superficial position of the stylomastoid foramen, care must be taken when defining the inferior border of the Palva flap as an overly aggressive approach in this region could damage the facial nerve trunk, which can be encountered about 1 cm anterior to the mastoid prominence in an infant (Farrior & Santini, 1985).

When performing mastoidectomy, it is critical to keep in mind the lower bone density and smaller dimensions of the mastoid in an infant patient. For example, bony maturation of the lateral cortex does not occur until around 1.7 years of age, and planimetry measurements of mastoid air cells at 1 year of age average between 3 and 4 cm^2^ compared with an average of 12 cm^2^ in adult patients (Takahashi et al., 2017). The mastoid antrum is fully developed at birth (Cinamon, 2009); therefore, drilling can typically be initiated just posterior to the bony external auditory canal (EAC) with care taken to define the tegmen mastoideum and sinodural angle. Infants younger than 12 months of age often have significant marrow within the developing mastoid (Sano et al., 2007); mastoidectomy in these regions can lead to significant bleeding, which must be promptly addressed to minimize blood loss. Application of topical epinephrine can slow bleeding, while bone wax can be effective at sealing off marrow or more brisk arterial and venous bleeding. In addition, liberal use of diamond burrs or drilling so that the burr spins in reverse rather than forward can assist with hemostasis. Despite the smaller dimensions of the mastoid bone, access is typically adequate for the facial recess to be performed in standard fashion. The facial recess dimensions in a newborn are approximately the same as in an adult, and therefore this is not a limitation to implanting infants younger than 12 months (Dahm et al., 1993; Eby, 1996).

If anatomy is favorable, electrode insertion can be performed using a round window approach. Alternatively, the electrode insertion can be performed via cochleostomy, usually just inferior to and at the level of the round window (peri round window), to control the angle of insertion within the confined working space of the infant mastoid (Cosetti & Roland, 2010b). A tight subperiosteal pocket can be prepared to accept the receiver-stimulator portion of the implant (Balkany et al., 2009; Jethanamest et al., 2014). Over time, implant surgery techniques have evolved to reduce the requirement for drilling a large well to recess the receiver-stimulator. Drilling a well can be more challenging in infants; because their skull is thin, dura may be exposed (Cohen et al., 2014). With the tight pocket technique, a minimal amount of subperiosteal elevation is performed to allow snug insertion of the receiver-stimulator under the posterior temporalis muscle. Surgeons may also choose to drill a shallow well to further inhibit migration (Miyamoto et al., 2017). Alternatively, studies have reported on the use of receiver-stimulator components with an additional small linear pedestal that can provide added retention similar to a well, but with minimal required drilling (Papsin et al., 2016; Parkes et al., 2017).

Children with cochleovestibular anomalies may experience egress of cerebrospinal fluid through the cochleostomy, but this can almost always be managed with fascial or periosteal packing after insertion of the electrode (Papsin, 2005). Following insertion, intraoperative audiometric evaluation can be helpful to ensure a well-functioning device and to guide postoperative mapping, which can be more challenging in young children. Options for testing may include impedance, electrically evoked stapedius reflex, neural response telemetry, and transimpedance measurements. Such measurements can help guide initial CI mapping in young children (Raghunandhan et al., 2014). Intraoperative audiometric monitoring can often be performed while the surgeon is closing the surgical site to avoid prolonging the duration of anesthetic. Finally, an anterior-posterior skull X-ray can be performed to confirm appropriate positioning of the electrode within the cochlea, verifying the insertion depth and absence of kinking or tip rollover (Fernandes et al., 2018); other X-ray views such as lateral or Stenver’s (Shaul et al., 2020) could be performed depending on surgeon preference.

## Conclusion

Congenitally deaf infants who are implanted prior to 12 months of age have the potential to develop near-normal expressive and receptive language skills. Infants are potentially candidates for cochlear implantation if they have been confirmed to have bilateral profound hearing loss and have intact auditory nerve anatomy to allow for effective electrical stimulation; however, CI candidacy assessment is multidisciplinary and must include audiometric, medical, communication, and family/environmental components. If there are inconsistencies that arise during the evaluation, these must be addressed prior to implantation. Surgeons performing infant implantation must recognize the specific challenges associated with mastoid surgery in children younger than 12 months.
